# Reply to “Comment on ‘Evaluation of the Long-Term Administration of Proton Pump Inhibitors (PPIs) in the Mineral Nutrient’s Bioavailability’”

**DOI:** 10.1021/acsomega.6c04757

**Published:** 2026-05-19

**Authors:** Andréa Santana de Brito, Angerson Nogueira do Nascimento, Fernando Luiz Affonso Fonseca, Alexandre Minami Fioroto, Giuliana Petri, Rafaela Garcia Vidigal do Nascimento

**Affiliations:** † Institute of Environmental, Chemical and Pharmaceutical Sciences (ICAQF), Federal University of São Paulo, CEP: 09972-270 São Paulo, Brazil; ‡ ABC Medical School, 125191 Faculty of Medicine of ABC—University Center (FMABC), CEP: 09060-870 Santo André, Brazil

## Abstract

This work presents a structured response to comments regarding the study “Evaluation of the Long-Term Administration of Proton Pump Inhibitors (PPIs) in the Mineral Nutrient’s Bioavailability”, published in *ACS Omega* ( de BritoA. S.,  ACS Omega 2025, 10, 56085−56095, 10.1021/acsomega.5c07700 41322596 PMC12658647). The comments raised by readers were carefully evaluated with reference to the original data and relevant scientific literature. The study was performed by using a controlled rat model with varying treatment durations. Physiological, biochemical, and hematological parameters as well as elemental concentrations were evaluated. The data were analyzed using appropriate statistical methods, and comparisons between the control and treated groups demonstrated measurable variations associated with PPI administration. These analyses indicate that the study suggests alterations in certain parameters that warrant increased public awareness regarding the long-term use of these medications. However, further investigations are necessary to confirm and expand upon the experimental findings observed in the animal model. The study did not assess vitamin B12 levels or cancer risk associated with the prolonged use of omeprazole. Therefore, claims regarding these outcomes were made without consultation with the authors and are not supported by the published data. A responsible interpretation of these findings requires careful consideration of the study’s scope and limitations, avoiding extrapolations that were not explicitly addressed in the original work.

The study published in *ACS Omega* entitled “Evaluation of the Long-Term Administration of Proton Pump Inhibitors (PPIs) in the Mineral Nutrient’s Bioavailability” (10.1021/acsomega.5c07700) aimed to investigate laboratory and mineral changes associated with omeprazole exposure in an experimental rat model, as well as its potential effects on the absorption and bioavailability of elements.

The experimental design included a controlled rat model with treatment durations of 10, 30, and 60 days. Physiological, biochemical, and hematological parameters were systematically evaluated, while elemental concentrations were quantified using inductively coupled plasma mass spectrometry (ICP-MS). The results were analyzed using two-way analysis of variance (ANOVA), with post hoc comparisons performed using Tukey’s test and statistical significance established at *p* < 0.05. Additionally, effect sizes were calculated using Cohen’s *d* and classified as trivial, small, moderate, or large. Based on these statistical evaluations, differences between the control and treated groups were identified, indicating measurable variations associated with the treatment.

The results suggest alterations in hematological markers, including reductions in red blood cells, hemoglobin, and hematocrit, alongside imbalances in iron (Fe), copper (Cu), and calcium (Ca) levels in the blood and organs. These findings suggest that prolonged PPI administration may affect the mineral bioavailability and hematological status in this experimental model.

The omeprazole dose was selected to approximate a human-equivalent dose of 40 mg of omeprazole for a 60 kg individual, consistent with established practices in rodent pharmacology.^1–3^ Due to differences in metabolic rate and drug clearance between rodents and humans, higher weight-adjusted doses are commonly required to achieve pharmacological effects comparable to those observed in humans. Furthermore, the purpose of the study was not to replicate the clinical therapeutic regimen, but rather to explore potential biological alterations associated with sustained acid suppression in a controlled experimental setting. The treatment duration was designed to allow sufficient time for gradual metabolic and hematological changes to emerge, particularly those related to mineral absorption and redistribution.

The dose used in this study is lower than those typically required to produce significant acid suppression in rodents, given that rodents metabolize PPIs more rapidly than humans. Then, this study represents a preliminary investigation conducted by our research group to evaluate the potential effects of omeprazole on mineral nutrient bioavailability. The experimental design will be refined to obtain more comprehensive information on the drug’s potential health effects. Furthermore, future studies will include different dose levels of omeprazole to determine whether higher or lower concentrations produce more pronounced responses than those observed in the present study.

It is important to emphasize that vitamin B12 levels and cancer risks were not evaluated in this study. These topics were not included in the study objectives and were not addressed in the original article. Therefore, interpretations suggesting that our work examined or established a relationship between proton pump inhibitor (PPI) use and vitamin B12 deficiency and cancer do not reflect the scope of the study. Furthermore, some media outlets are attributing information to the study without consulting or interviewing the authors, and these interpretations do not reflect the conclusions presented in the original article.

Regarding the absence of specific markers of anemia, the diagnosis of anemia is based on laboratory markers that determine whether anemia is present and then helps identify its type and underlying cause. The most important markers used to confirm anemia are hemoglobin, hematocrit, and the red blood cell (RBC) count. Hemoglobin is the protein in red blood cells responsible for transporting oxygen throughout the body; when hemoglobin levels are low, the blood’s capacity to carry oxygen decreases, which is the central feature of anemia. Hematocrit measures the proportion of blood volume that consists of red blood cells; therefore, a low hematocrit level indicates that the percentage of red blood cells in the blood is reduced. Similarly, the RBC count measures the number of red blood cells present in each volume of blood, and a decreased count often accompanies anemia. Together, these parameters indicate whether the subject has a reduced oxygen-carrying capacity of the blood and therefore suggest the presence of anemia. The term “suggest” was frequently used in this publication to indicate that the data or results point toward a possible conclusion but do not provide definitive proof, meaning that further studies are required to advance knowledge in this area.

Animal models remain essential tools for investigating biological mechanisms, even though substantial physiological differences among species prevent direct extrapolation. However, these models remain an essential step in biomedical investigation, particularly for exploring biological mechanisms that cannot be directly assessed in humans.

With respect to the literature on PPIs and bone health, there are studies suggesting an association between PPI use and increased fracture risk.^4–8^ However, the relationship with bone mineral density changes remains complex and requires further investigation.^4–8^

Regarding hypomagnesemia, the literature provides evidence that PPIs can cause hypomagnesemia, particularly with long-term therapy, in patients taking diuretics, in those with renal impairment, or in older individuals. This effect is reversible upon PPI discontinuation.^9–18^

Concerning vitamin B12, PPI use has been associated with lower vitamin B12 levels in some studies, although the relationship is inconsistent across the literature.^19–24^ However, it is important to clarify that vitamin B12 levels were not assessed in our study. Any claims suggesting otherwise may stem from secondary sources or media interpretations, rather than from the original publication in *ACS Omega* (10.1021/acsomega.5c07700), which did not report such measurements.

Furthermore, the relationship between PPIs and cancer risk has been a subject of extensive research, with evidence varying across different publications.^25–29^ Some studies suggest an increased risk, while others find no significant association.^25–29^ International guidelines reinforce that PPIs are safe when used according to appropriate clinical indications. However, we emphasize that our study does not mention cancer causation in humans. Therefore, any such claim is not supported by our data and misrepresents the findings of our study. It is possible that some readers are relying on secondary sources that did not accurately represent the authors’ intended conclusions.

In conclusion, the recent study published in *ACS Omega* (10.1021/acsomega.5c07700) rigorously reports only the observed differences between the control and treated groups within a controlled animal model. The publication consistently recommended that future research investigate the long-term effects of omeprazole over extended durations and with refined methodologies. Responsible interpretation of the data requires acknowledging the inherent limitations of preclinical models and underscores the critical need for confirmatory clinical studies to fully assess the long-term risks and benefits of PPI use in human populations.
